# Limiting prolonged inflammation during proliferation and remodeling phases of wound healing in streptozotocin-induced diabetic rats supplemented with camel undenatured whey protein

**DOI:** 10.1186/1471-2172-14-31

**Published:** 2013-07-25

**Authors:** Hossam Ebaid, Osama M Ahmed, Ayman M Mahmoud, Rasha R Ahmed

**Affiliations:** 1 Department of Zoology, College of Science, King Saud University, P.O. Box 2455, Riyadh, 11451, Saudi Arabia; 2 Department of Zoology, Faculty of Science, El-Minia University, Minya, Egypt; 3 Physiology Division, Zoology Department, Faculty of Science, Beni-Suef University, Beni-Suef, Egypt; 4 Cell Biology and Histology Division, Zoology Department, Faculty of Science, Beni-Suef University, Beni-Suef, Egypt

**Keywords:** Whey proteins, Prolonged inflammation, Cytokines, Wound healing, Diabetic rats

## Abstract

**Background:**

Impaired diabetic wound healing occurs as a consequence of excessive reactive oxygen species (ROS) and inflammatory cytokine production. We previously found that whey protein (WP) was able to normally regulate the ROS and inflammatory cytokines during the inflammatory phase (first day) in streptozotocin (STZ)-diabetic wound healing. This study was designed to assess the effect of WP on metabolic status, the inflammation and anti-inflammation response, oxidative stress and the antioxidant defense system during different phases of the wound healing process in diabetic rats. WP at a dosage of 100 mg/kg of body weight, dissolved in 1% CMC, was orally administered daily to wounded normal (non-diabetic) and STZ-induced diabetic rats for 8 days starting from the 1^st^ day after wounding.

**Results:**

The data revealed that WP enhanced wound closure and was associated with an increase in serum insulin levels in diabetic rats and an alleviation of hyperglycemic and hyperlipidemic states in diabetic animals. The increase in insulin levels as a result of WP administration is associated with a marked multiplication of β-cells in the core of islets of Langerhans. WP induced a reduction in serum TNF-α, IL-1β and IL-6 levels and an increase in IL-10 levels, especially on the 4^th^ day after wounding and treatment. WP also suppressed hepatic lipid peroxidation and stimulated the antioxidant defense system by increasing the level of glutathione and the activity of glutathione-S-transferase, glutathione peroxidase and superoxide dismutase (SOD) in wounded diabetic rats.

**Conclusions:**

WP was observed to enhance wound closure by improving the diabetic condition, limiting prolonged inflammation, suppressing oxidative stress and elevating the antioxidant defense system in diabetic rats.

## Background

Diabetes is a complex metabolic disorder involving many organs and can devastate the lives of affected individuals [1]. Impaired wound healing is a complication of diabetes and a serious problem in clinical practice [2]. As many as 15% of people with diabetes will develop foot ulceration and wounds, and 3% will require lower limb amputation [3]. In addition, an increased incidence of wound complications in surgical patients with diabetes mellitus increases the general surgical risks due to the metabolic abnormalities associated with diabetes mellitus [4].

In humans and in all mammalian species, the wound healing process can be subdivided into distinct consecutive and overlapping stages: hemostasis, inflammation and debridement, new tissue formation and remodeling [5,6]. Neutrophils and macrophages play a key role in the inflammation phase of wound repair by secreting cytokines and a variety of growth factors [7].

Oxidative stress occurs due to an imbalance between the production of reactive oxygen species (ROS) and protection by cellular anti-oxidants [8]. Increased production of ROS and decreased antioxidant defense can cause tissue injury or even cell death, which can occur by necrosis and apoptosis [9]. Oxidative stress has been implicated in the pathology of diabetes mellitus [10], a disease marked by a prolonged inflammatory period that increases the time required for recovery. In addition, Mohammad et al. [11] stated that impaired wound healing occurs as a consequence of excessive ROS production. Thus, elimination of reactive oxygen species is an important strategy to improve the healing of wounds in diabetes mellitus patients.

Whey protein (WP) contains all of the essential and nonessential amino acids and is a good source of glutamine and the branched-chain amino acids that are necessary for cell growth [12]. The healing of bone, skin and muscle tissue is stimulated by the branch-chain amino acids leucine, isoleucine and valine. The amino acid proline aids in the production of collagen, thereby healing cartilage and strengthening joints, tendons and cardiac muscle [13]. In addition, WP has been found to significantly suppress hydroperoxide and ROS levels in leukocytes, liver and cutaneous tissues in mice by stimulating production of the antioxidant glutathione [14]. In addition, we previously observed that the in vitro chemotaxis of B cells, T cells and bone marrow-derived dendritic cells toward CCL-21 and CXCL-12 was significantly increased after WP administration [15].

Data from our previous work indicate that WP increases the capacity of non-diabetic [16] and diabetic [17] animals to heal wounds. That study revealed the potential effects of WP on immune processes, including the regulation of cytokines during inflammatory phase of healing process. Here, after inducing diabetes, we followed oxidative stress and sequential inflammatory and anti-inflammatory cytokines, and metabolic state to address whether WP can be able to limit the diabetic prolonged inflammation during proliferation and remodeling phases of cutaneous wound healing.

## Methods

### Preparation of whey proteins

Camel milk was obtained from a camel breed (Majaheem) from the Najd region in Saudi Arabia. The milk was skimmed by centrifugation at 5000 g for 20 min using an IEC Model K centrifuge [Boston, USA]. Skim milk was acidified to pH 4.3 using 1 M HCl. The precipitated casein was removed by centrifugation, and the supernatant containing the whey protein was saturated with ammonium sulfate (70% saturation) and incubated overnight at 4°C. The precipitated whey protein was collected by centrifugation and dialyzed against distilled water for 48 h at 4°C using a Spectra/Pro® Membrane, MWCO 6000-8000 Da. The obtained dialyzate was lyophilized using a Unitop 600SL, [Virtis Company, Gardiner, New York 12525 USA] and were kept at -20°C until use. The dialyzate containing non-denatured whey protein was freeze-dried and refrigerated until use.

### Ethical approval

All animal procedures were conducted in accordance with the standards set forth in the guidelines for the care and use of experimental animals by the Committee for the Purpose of Control and Supervision of Experiments on Animals (CPCSEA) and the National Institutes of Health (NIH). The study protocol was approved by the Animal Ethics Committee of the Zoology Department in the College of Science at Beni-Suef University.

### Experimental animals

Male albino rats (*Rattus rattus*) weighing approximately 150-180 g were used as experimental animals in this study. The animals were housed in standard polypropylene cages with aerated, stainless steel covers, maintained at a controlled temperature (22 ± 2°C) with a 12 h light/dark cycle and were fed a standard diet of known composition with water ad libitum.

### Diabetic models

Diabetes was induced by a single injection of freshly dissolved STZ (50 mg/kg of body weight; Sigma, USA) in a 0.1 mol/l citrate buffer (pH 4.5) into the peritoneum [18]. Control rats were injected with citrate buffer. Seven days after STZ injection, rats were screened for serum glucose levels. Rats with a serum glucose level ≥ 200 mg/dl after 2 hours of glucose intake were considered diabetic and selected for further studies.

### Experimental design

The supplemented volume for all groups was constant and did not exceed 250 μl per dosage per day. The optimal dose of WP was determined in our laboratory on the basis of the LD_50_ and several established studies and parameters. The animals were allocated into 6 groups of 12 animals each, assigned as follows:

1. Uninjured control group (Non-wounded normal: Non-wounded N) that were orally supplemented with distilled water (250 μl/mouse/day).

2. Wounded non-diabetic group with daily administration of the vehicle (250 μl/mouse/day), 1% carboxymethyl cellulose (CMC), by gastric intubation for 4 days (n = 6) or by gastric intubation for 8 days (n = 6) (Wounded normal: N).

3. Wounded non-diabetic group with daily administration of WP at 100 mg/kg of body weight (250 μl/mouse/day), dissolved in 1% CMC, by gastric intubation either for 4 days (n = 6) or for 8 days (n = 6) (WP-treated wounded normal: WPN).

4. Uninjured diabetic group (Non-wounded diabetic: Non-wounded D) that were orally supplemented with distilled water (250 μl/mouse/day).

5. Wounded diabetic group with daily administration of 1% CMC (250 μl/mouse/day) by gastric intubation for 4 days (n = 6) or by gastric intubation for 8 days (n = 6) (Wounded diabetic: D).

6. Wounded diabetic group with daily treatment of WP at 100 mg/kg of body weight (250 μl/mouse/day) by gastric intubation either for 4 days (n = 6) or for 8 days (n = 6) (WP-treated wounded diabetic: WPD).

### Excisional wound preparation

Rats were anesthetized, and the back of the rat was shaved and sterilized using an alcohol swab. The wound biopsy model used in this experiment was performed as previously described [19] with slight modification. The shaved skin was pinched and folded, and the wound was punched through the full thickness of the folded skin to form a 2 × 5 mm rectangle below the shoulder blades of each rat.

Wounds from individual rats were digitally photographed every day. A standard rectangle equivalent in size to the initial wound area was drawn beside the wound and used as a reference. Wound size was calculated by determining the area of the wound each day in comparison to the area of the standard rectangle. Wound closure was expressed as the ratio of the initial wound size to the wound area (each day after wounding). A higher ratio indicates faster wound closure.

### Blood and tissue sampling

At the end of the experiment, animals were sacrificed, and blood samples were obtained from the carotid artery. Then, animals were decapitated and dissected, and livers as well as pancreases of were rapidly excised. Blood samples were left to coagulate and then were centrifuged, and clear non-hemolyzed serum was kept at -20ºC until used. Livers were kept at -20ºC pending homogenization while pancreases were fixed in neutral buffered formalin and transferred to Pathology Department, National Cancer Institute (Cairo, Egypt) for blocking, sectioning and staining with modified aldehyde fuchsin method [20].

### Estimation of cytokines

The levels of TNF-α and IL-10 in the serum of control and experimental groups were determined using specific ELISA kits purchased from R and A Systems, USA. Serum IL-1β and IL-4 ELISA kits were obtained from Thermo Scientific (USA) and Invitrogen (Canada). The concentrations of TNF-α, IL-1β, IL-4 and IL-10 were determined using a spectrophotometer at 450 nm according to the manufacturers’ instructions. Standard plots were constructed using standards, and the concentrations for unknown samples were calculated from the standard plot.

### Estimation of glucose concentration

Serum glucose concentration was determined according to the Trinder method [21] using a commercial diagnostic kit (Biodiagnostics, Egypt). Serum insulin was assayed in the Radioactive Isotopes Unit, Middle Eastern Regional Radioisotope Center (Dokki, Giza) using a DPC radioimmunoassay kit (Diagnostic Products Corporation, Los Angeles, USA) [coat-A-count] according to the method reported by Marschner et al. [22].

### Estimation of serum lipids

Serum triglycerides [23], cholesterol [24] and HDL-cholesterol [25] were estimated using a reagent kit (Biodiagnostics, Egypt). The serum LDL-cholesterol level was calculated using the Friedewald [26] formula (LDL cholesterol = total cholesterol – triglycerides/5 – HDL cholesterol). The serum vLDL-cholesterol concentration was calculated according to the Nobert [27] formula (vLDL-cholesterol = triglycerides/5). Cardiovascular risk indices were calculated by dividing the total cholesterol and the LDL-cholesterol level by the HDL-cholesterol level.

### Oxidative stress parameters

Liver lipid peroxidation, reduced glutathione, superoxide dismutase and peroxidase activities were also measured according to the methods of Preuss et al. [28], Beutler et al. [29], Marklund & Marklund [30] and Kar & Mishra [31], respectively. The total antioxidant levels in serum were assayed as previously described Koracevic et al. [32] using a reagent kit purchased from Biodiagnostics (Egypt).

### Statistical analysis

The data were analyzed using a one-way analysis of variance (ANOVA) (PC-STAT, University of Georgia, 1985) followed by the LSD test to compare various groups with each other. The results were expressed as the mean ± SE, and values of P > 0.05 were not considered significantly different, whereas values of P < 0.05 and P < 0.01 were considered significant and highly significant, respectively.

## Results

Wound healing requires a prolonged period of time in diabetic rats. The results indicated that the time required to heal wounds was significantly decreased in diabetic rats supplemented with WP compared with diabetic rats treated with vehicle.

### Effect of WP on the islets' β-cells

**Figure 1:**
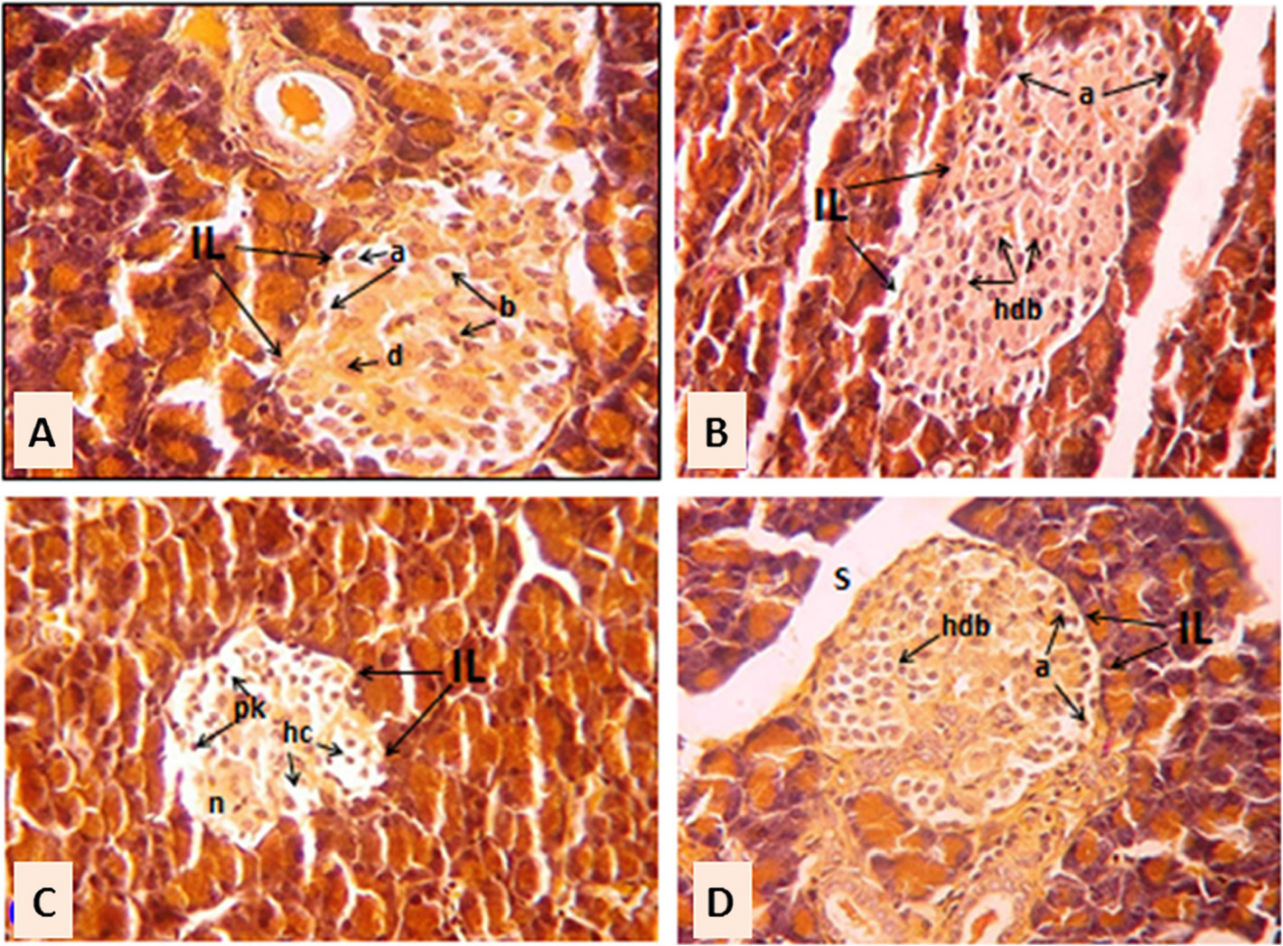
**Photomicrographs of pancreas sections of normal wounded control. (A)**, normal wounded rats treated with WP for 8 days **(B)**, diabetic wounded control **(C)** and diabetic wounded rats treated with WP for 8 days **(D)**. The increase in insulin levels as a result of WP administration is associated with a marked multiplication of β-cells in the core of islets of Langerhans. IL: islets of Langerhans; a: alpha cells; b: beta cells; d: delta cells; hdv: highly divided beta cells; S: septae separating pancreatic lobules; hc: hydropic cells; n: necrosis; pk: pyknotic nuclei (Modified aldehyde fuchsin method; 400x).

The photomicgraphs of pancreatic sections illustrated that the administration of WP to wounded normal rats for 8 days augmented the multiplication of islets' cell. The islets have larger size with increased number of granulated β-cells which seemed to have a higher division rate as compared to normal wounded control. On the other hand, the diabetic wounded rats exhibited marked decrease in the number of islets' cells associated with hydropic degenerations and pyknosis in the islets. The administration of WP to the diabetic wounded rats for 8 days produced a potential improvement in the islets. The β-cells seemed highly proliferated and densely granulated (Figure 1).

### Effects of WP on the insulin and glucose levels, and wound closure

**Figure 2:**
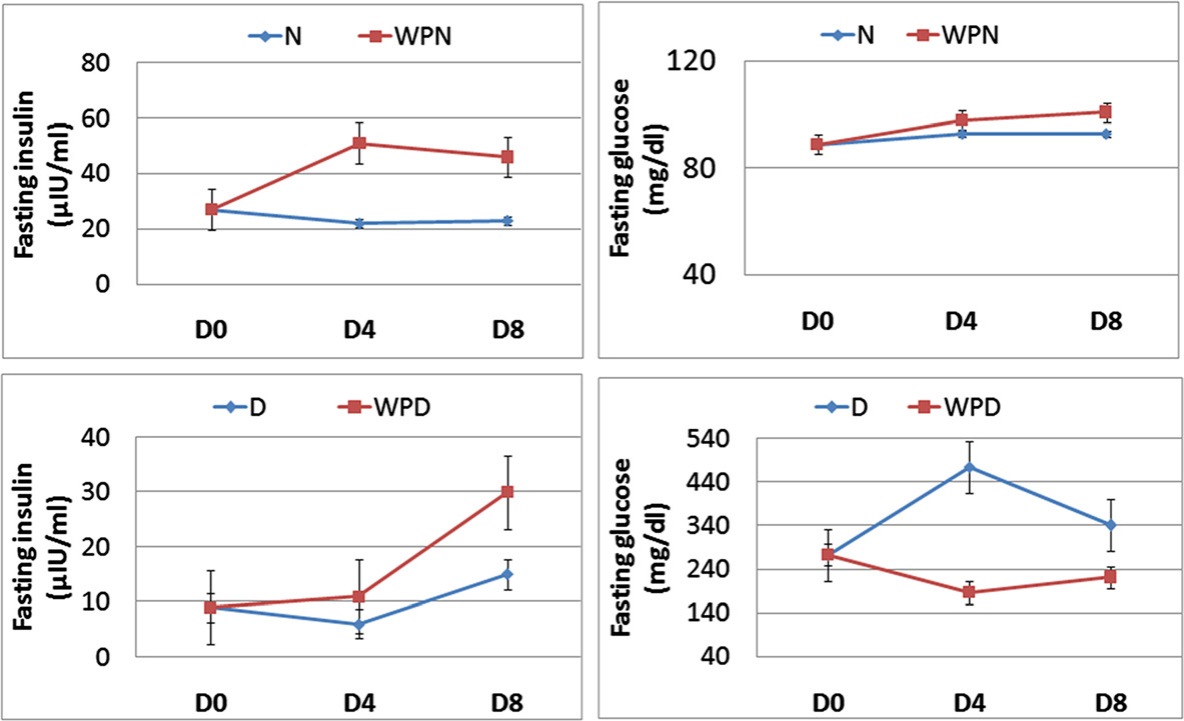
**Effect of whey protein administration on serum glucose and insulin concentrations of wounded normal and wounded streptozotocin-induced diabetic rats.** Oral administration of WP to wounded diabetic rats for 4 or 8 days potentially induced amelioration of the elevated serum glucose levels. The serum insulin concentration was markedly increased as a result of treating wounded non-diabetic and diabetic rats for 4 and 8 days. Non-wounded N; wounded normal: N; Whey protein-treated wounded normal: WPN; Non-wounded diabetic: Non-wounded D; Wounded diabetic: D; Whey protein-treated wounded diabetic: WPD.

**Figure 3:**
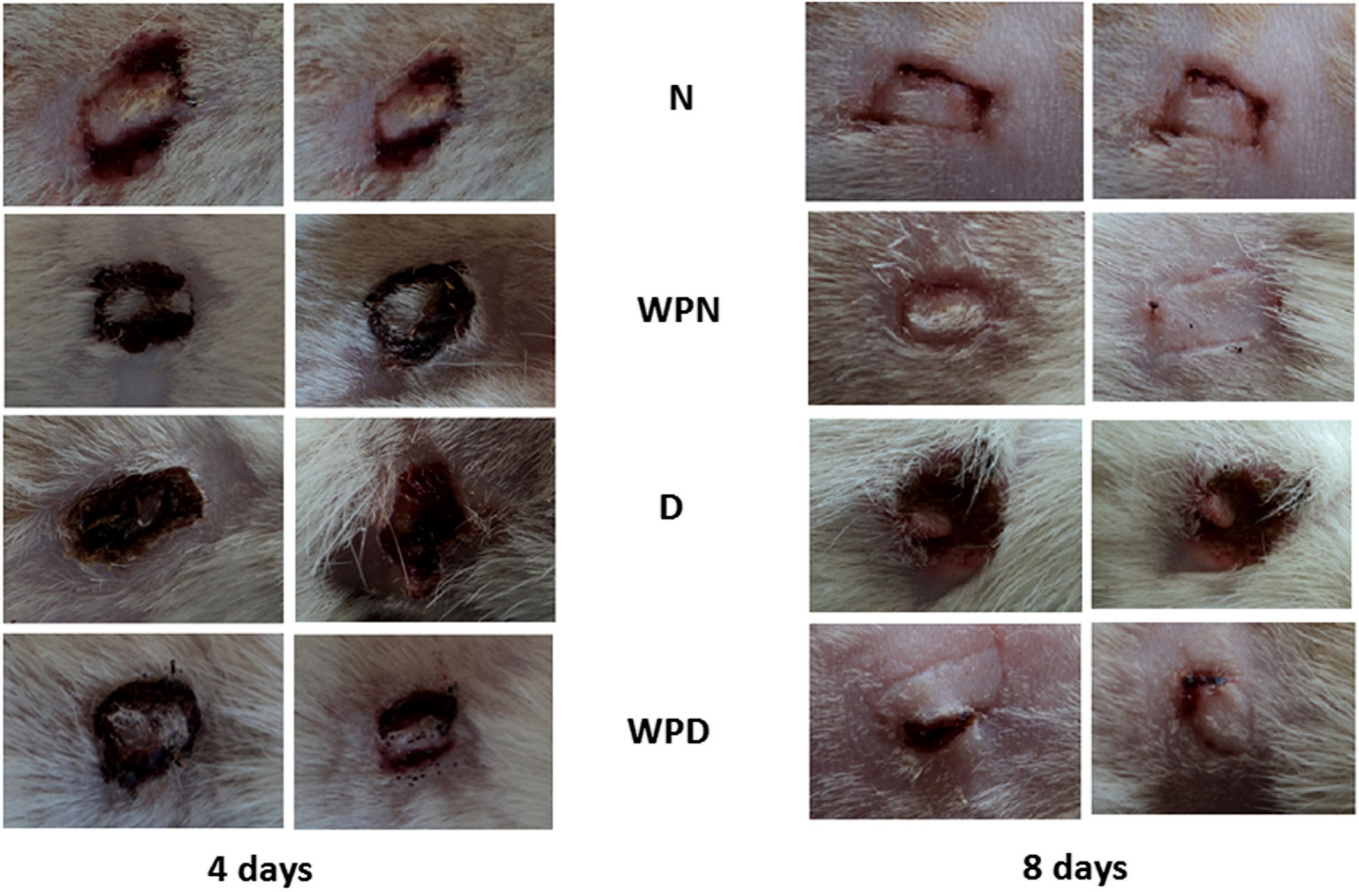
**Photograph of external cutaneous wound healing process in control and different treated groups.** Whey protein enhanced wound closure in diabetic models. This figure reveals that WP enhanced wound closure process in non-diabetic and diabetic rats. Non-wounded N; wounded normal: N; Whey protein-treated wounded normal: WPN; Non-wounded diabetic: Non-wounded D; Wounded diabetic: D; Whey protein-treated wounded diabetic: WPD.

The effect of WP on serum fasting glucose and insulin levels is depicted in Figure 2. Serum glucose concentration was not significantly affected in wounded non-diabetic rats regardless of WP treatment on the 4^th^ and 8^th^ day after wounding. It was very significantly elevated (p < 0.01; LSD) in uninjured and wounded diabetic rats compared with control rats. Oral administration of WP to wounded diabetic rats for 4 or 8 days potentially induced amelioration (p < 0.01; LSD) of the elevated serum glucose levels. The serum insulin concentration, on the other hand, was markedly increased as a result of treating wounded non-diabetic and diabetic rats for 4 and 8 days, compared with the corresponding untreated wounded and uninjured groups. The enhancement of insulin level was more pronounced if the treatment was extended from 4 to 8 days. A one-way analysis of variance (ANOVA) indicated that the effect between groups on serum glucose and C-peptide is highly significant (p < 0.001; F-prob.) throughout the experiment (Figure 2). All wounded diabetic animals treated with WP achieved complete healing by day 8 (Figure 3).

### Effects of WP on the serum lipid profile

The serum lipid profile, including triglycerides (TG), total cholesterol (TC), LDL-cholesterol (LDL-C), HDL-cholesterol (HDL-C) and vLDL-cholesterol (vLDL-C) as well as the cardiovascular risk indices represented by the ratios of TC and LDL-C to HDL-C are represented in Table 1. The lipid profile and the cardiovascular risk indices were not significantly affected by wounding or by WP treatment of wounded non-diabetic and diabetic rats. The uninjured and wounded diabetic rats exhibited a significant increase in the levels of TG, TC, LDL-C and vLDL-C as well as the ratios of TC and LDL-C to HDL-C compared with control rats. The treatment of wounded diabetic rats with WP for 4 and 8 days induced potential amelioration of the altered parameters. One-way ANOVA indicated that the effect on serum TG, TC, vLDL-C and LDL-C/HDL-C levels between groups was highly significant (p < 0.01; F-prob.), and the effect on serum LDL-C and TC/HDL-C was very highly significant (p < 0.001) (Table 1).

**Table 1:** Effect of whey protein administration on serum lipid profile of wounded normal and wounded streptozotocin-induced diabetic rats

**Days post-wounding**	**Triglycerides (mg/dl)**	**Cholesterol (TC) (mg/dl)**	**LDL-C (mg/dl)**	**HDL-C (mg/dl)**	**vLDL-C (mg/dl)**	**LDL-C/HDL**	**TC/HDL-C**
Non-wounded N	48.46 ± 3.62^c^	53.59 ± 3.67^c^	20.76 ± 2.05^d^	33.87 ± 1.24^a^	9.70 ± 0.72^c^	0.61 ± 0.44^c^	1.61 ± 0.04^d^
N (4 days)	47.01 ± 3.35^c^	56.89 ± 8.15^c^	20.80 ± 2.71^d^	33.74 ± 2.06^a^	9.41 ± 0.67^c^	0.65 ± 0.06^c^	1.73 ± 0.07^d^
N (8 days)	62.91 ± 2.56^c^	46.08 ± 4.87^c^	17.45 ± 1.36^d^	25.49 ± 3.62^a^	12.58 ± 0.51^c^	0.72 ± 0.06^bc ^	1.78 ± 0.05^d^
WPN (4 days)	65.19 ± 2.06^c^	64.59 ± 4.61^bc^	23.55 ± 1.74^cd^	37.78 ± 5.71^a^	13.04 ± 0.41^c^	0.63 ± 0.09^c^	1.79 ± 0.20^d^
WPN (8 days)	69.23 ± 9.05^c^	57.04 ± 8.86^c^	22.61 ± 1.53^d^	31.84 ± 3.42^a^	13.30 ± 1.81^c^	0.76 ± 0.06^bc^	1.75 ± 0.08^d^
Non-wounded D	87.08 ± 6.72^bc^	85.89 ± 6.78^ab^	38.80 ± 3.48^a^	32.53 ± 0.96^a^	17.26 ± 1.68^bc^	1.25 ± 0.10^a^	2.55 ± 0.13^a^
D (4 days)	160.59 ± 16.69^ab^	85.59 ± 4.56^ab^	38.19 ± 3.31^ab^	39.43 ± 3.21^a^	32.12 ± 3.34^ab^	1.00 ± 0.12^ab^	2.20 ± 0.11^bc^
D (8 days)	90.43 ± 3.27^bc^	53.11 ± 5.28^c^	18.98 ± 0.87^d^	29.56 ± 4.59^a^	18.32 ± 0.63^bc^	0.64 ± 0.11^c^	1.88 ± 0.09^cd^
WPD (4 days)	219.51 ± 64.57^a^	88.05 ± 8.73^a^	36.70 ± 3.06^ab^	45.53 ± 1.76^a^	43.91 ± 12.91^a^	0.82 ± 0.12^bc^	2.33 ± 0.16^ab^
WPD (8 days)	84.33 ±7.60^bc^	53.75 ± 5.96^c^	16.83 ± 2.13^d^	32.69 ± 3.43^a^	16.87 ± 1.52^bc^	0.71 ± 0.10^bc^	1.87 ± 0.12^cd^
F-probability	p < 0.01	p < 0.01	p < 0.001	p > 0.05	p < 0.01	p < 0.01	p < 0.001
LSD at the 5% level	7.68	22.67	8.41	-	15.34	0.32	0.34
LSD at the 1% level	10.34	30.53	11.32	-	20.66	0.43	0.46

The means, which share the same superscript symbol(s) are non-significantly different.

### Regulation of TNF-α, IL-1β, IL-6 and IL-10 by supplementation with WP

**Figure 4:**
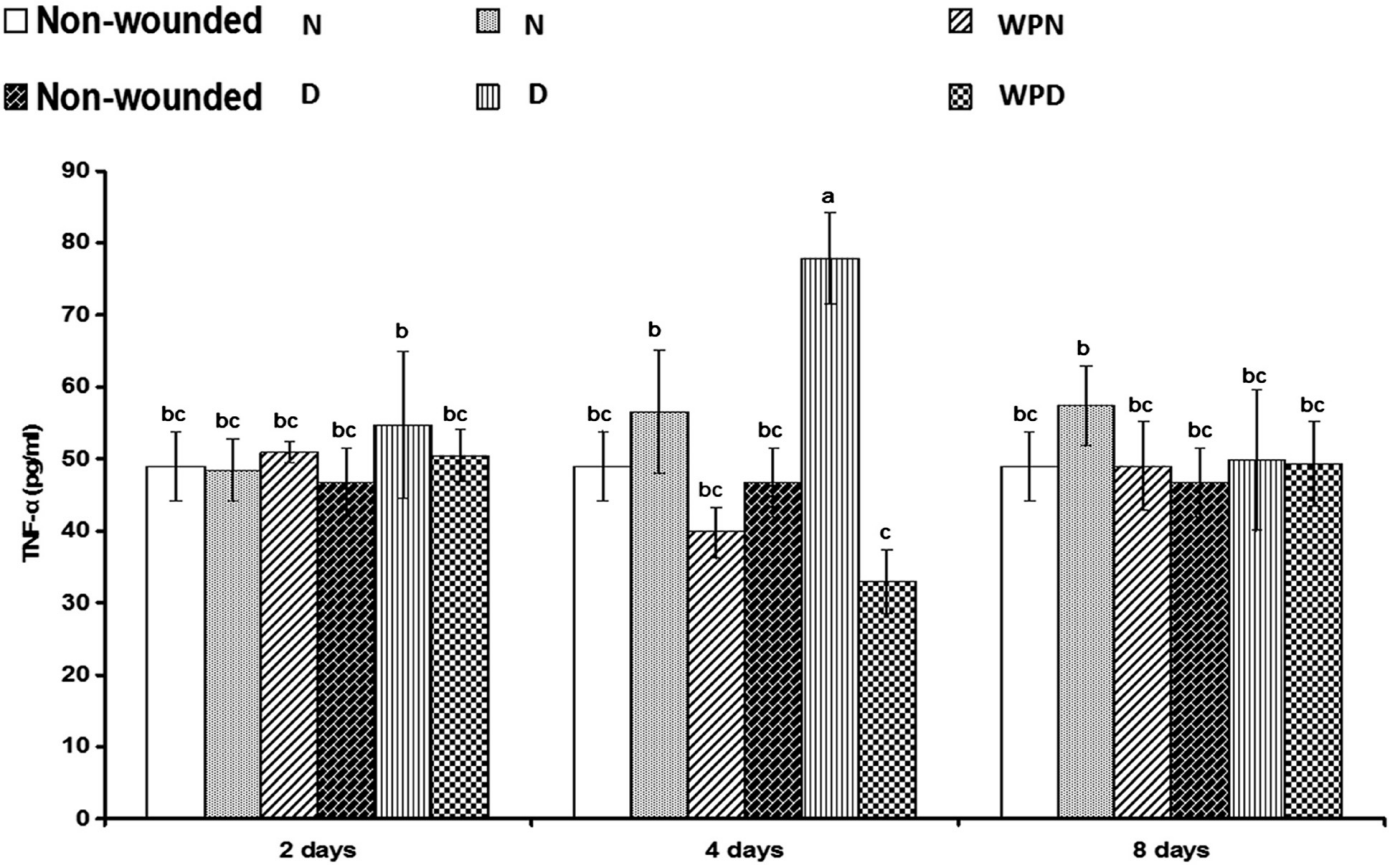
**Effect of whey protein administration on serum TNF-α concentration in wounded normal and wounded diabetic rats.** WP induced a reduction in serum TNF-α level especially on the 4^th^ day after wounding and treatment. Non-wounded N; wounded normal: N; Whey protein-treated wounded normal: WPN; Non-wounded diabetic: Non-wounded D; Wounded diabetic: D; Whey protein-treated wounded diabetic: WPD. F. Prob.: p < 0.01; LSD at the 5% level: 17.72; LSD at the 1% level: 23.86. The means, which share the same superscript symbol(s) are non-significantly different.

**Figure 5:**
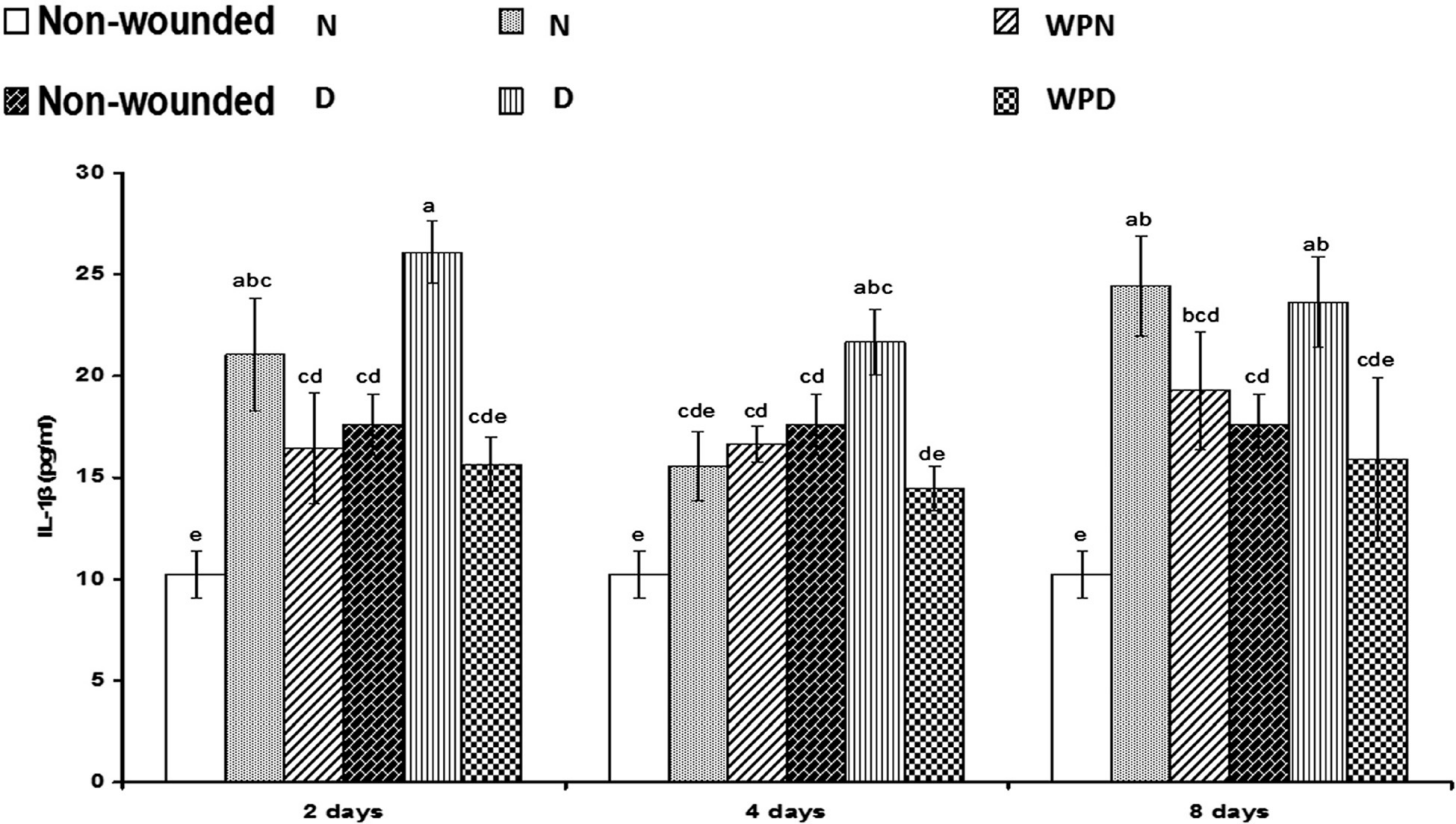
**Effect of whey protein administration on serum IL-1β concentration in wounded normal and wounded diabetic rats.** WP was found to induce a reduction in serum IL-1β level on the 4^th^ day after wounding and treatment. Non-wounded N; wounded normal: N; Whey protein-treated wounded normal: WPN; Non-wounded diabetic: Non-wounded D; Wounded diabetic: D; Whey protein-treated wounded diabetic: WPD. F. Prob.: p < 0.001; LSD at the 5% level: 5.99; LSD at the 1% level: 8.07. The means, which share the same superscript symbol(s) are non-significantly different.

**Figure 6:**
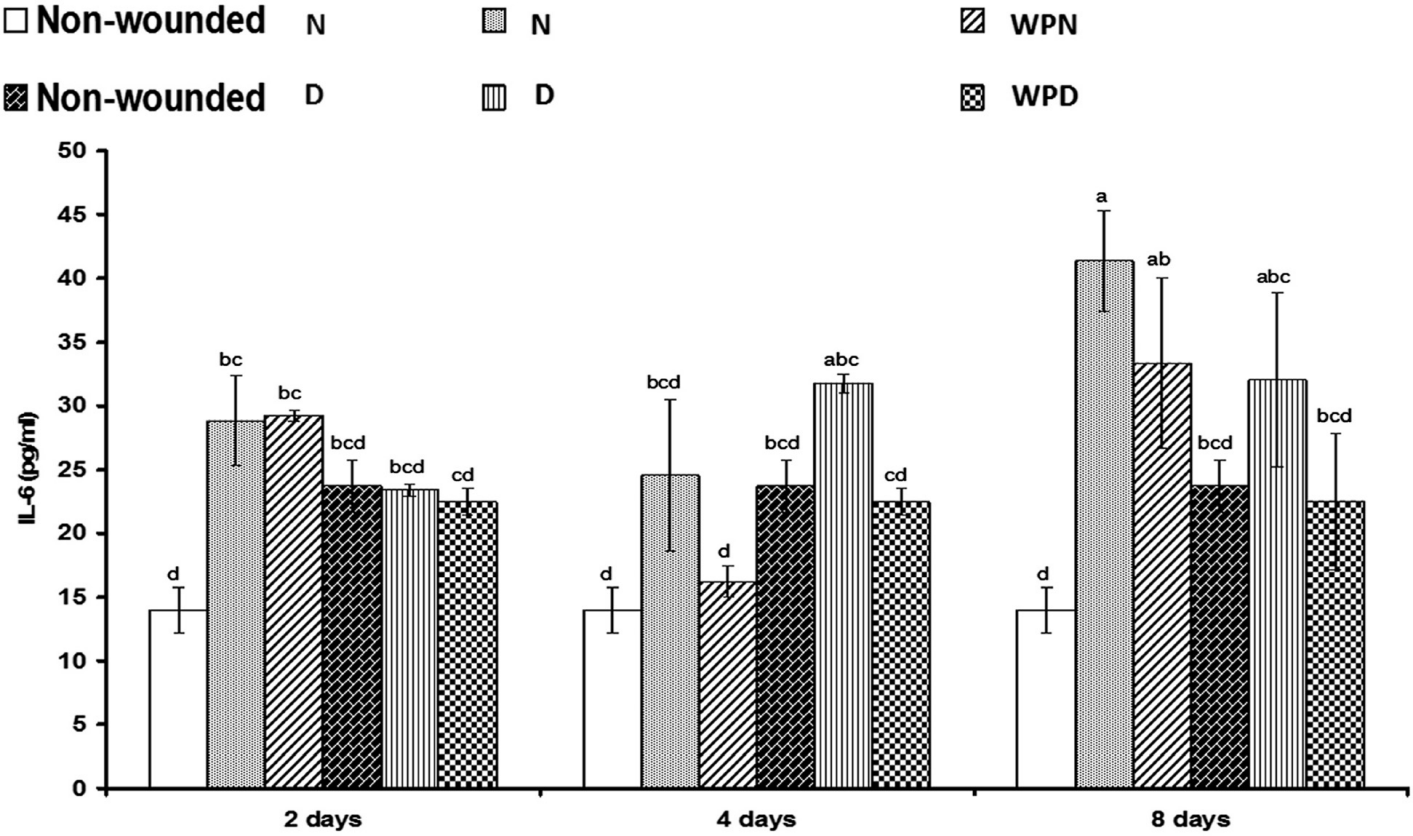
**Effect of whey protein administration on serum IL-6 concentration in wounded normal and wounded diabetic rats.** WP induced a reduction in serum IL-6 levels and on the 4^th^ day after wounding and treatment. Non-wounded N; wounded normal: N; Whey protein-treated wounded normal: WPN; Non-wounded diabetic: Non-wounded D; Wounded diabetic: D; Whey protein-treated wounded diabetic: WPD. F. Prob.: p < 0.001; LSD at the 5% level: 10.86; LSD at the 1% level: 14.62. The means, which share the same superscript symbol(s) are non-significantly different.

**Figure 7:**
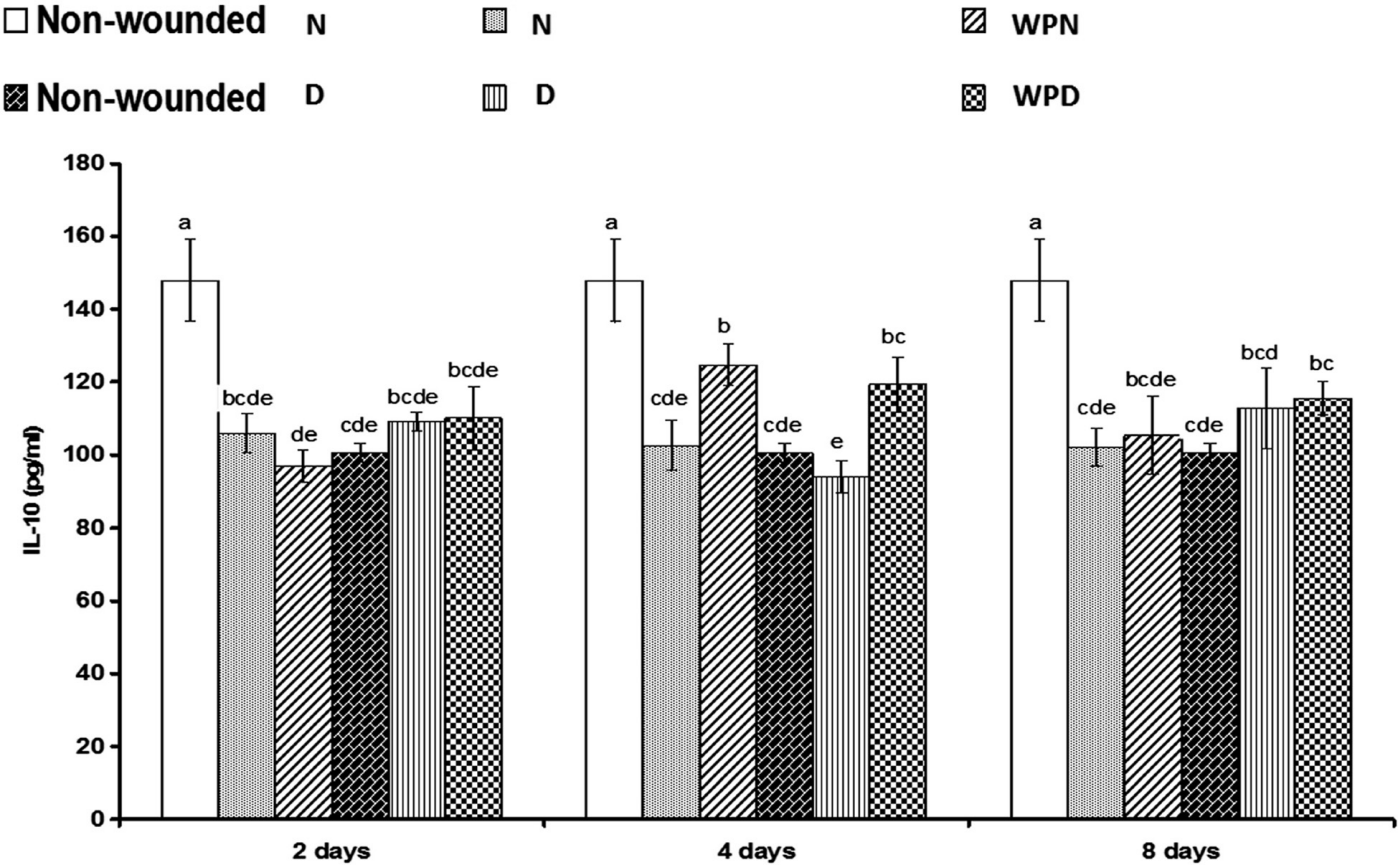
**Effect of whey protein administration on serum IL-10 concentration in wounded normal and wounded diabetic rats.** WP induced an increase in serum IL-10 levels, especially on the 4^th^ day after wounding and treatment. Non-wounded N; wounded normal: N; Whey protein-treated wounded normal: WPN; Non-wounded diabetic: Non-wounded D; Wounded diabetic: D; Whey protein-treated wounded diabetic: WPD. F. Prob.: p < 0.001; LSD at the 5% level: 20.37; LSD at the 1% level: 27.44. The means, which share the same superscript symbol(s) are non-significantly different.

Serum TNF-α levels were only significantly (p < 0.01; LSD) increased in wounded diabetic rats 4 days after wounding but were not significantly (p > 0.05; LSD) increased in wounded non-diabetic rats compared with the corresponding uninjured control (Figure 4). The treatment of wounded diabetic rats with WP for 4 days successfully decreased (p < 0.01; LSD) the elevated TNF-α level to values close to normal. Serum IL-1β levels were detectably increased in wounded non-diabetic and wounded diabetic rats compared with the corresponding uninjured control (Figure 5); serum IL-1β levels were significantly elevated in wounded non-diabetic and wounded diabetic rats on the 2^nd^ and 8^th^ day after wounding. The treatment of wounded non-diabetic rats with WP produced an insignificant decrease (p > 0.05; LSD), whereas the treatment of wounded diabetic rats induced a significant decrease in the elevated IL-1β concentration. Similarly, serum IL-6 levels were profoundly increased in wounded non-diabetic and wounded diabetic rats. The treatment of wounded animals for 2 days produced no change in IL-6 concentration but induced a profound decrease on the 4^th^ and 8^th^ day compared with the corresponding wounded controls (Figure 6). By contrast, serum IL-10 levels were significantly decreased in wounded non-diabetic and diabetic rats. The administration of WP to wounded non-diabetic and diabetic rats induced a significant decrease (p < 0.05; LSD) of IL-10 levels on the 4^th^ day after wounding (Figure 7). One-way ANOVA revealed that the effect between groups was highly significant for the serum TNF-α levels (p < 0.01; F-prob.) but was very highly significant (p < 0.001) for IL-1β, IL-6 and IL-10 levels.

### Stimulating oxidative stability by supplementation with WP

**Figure 8:**
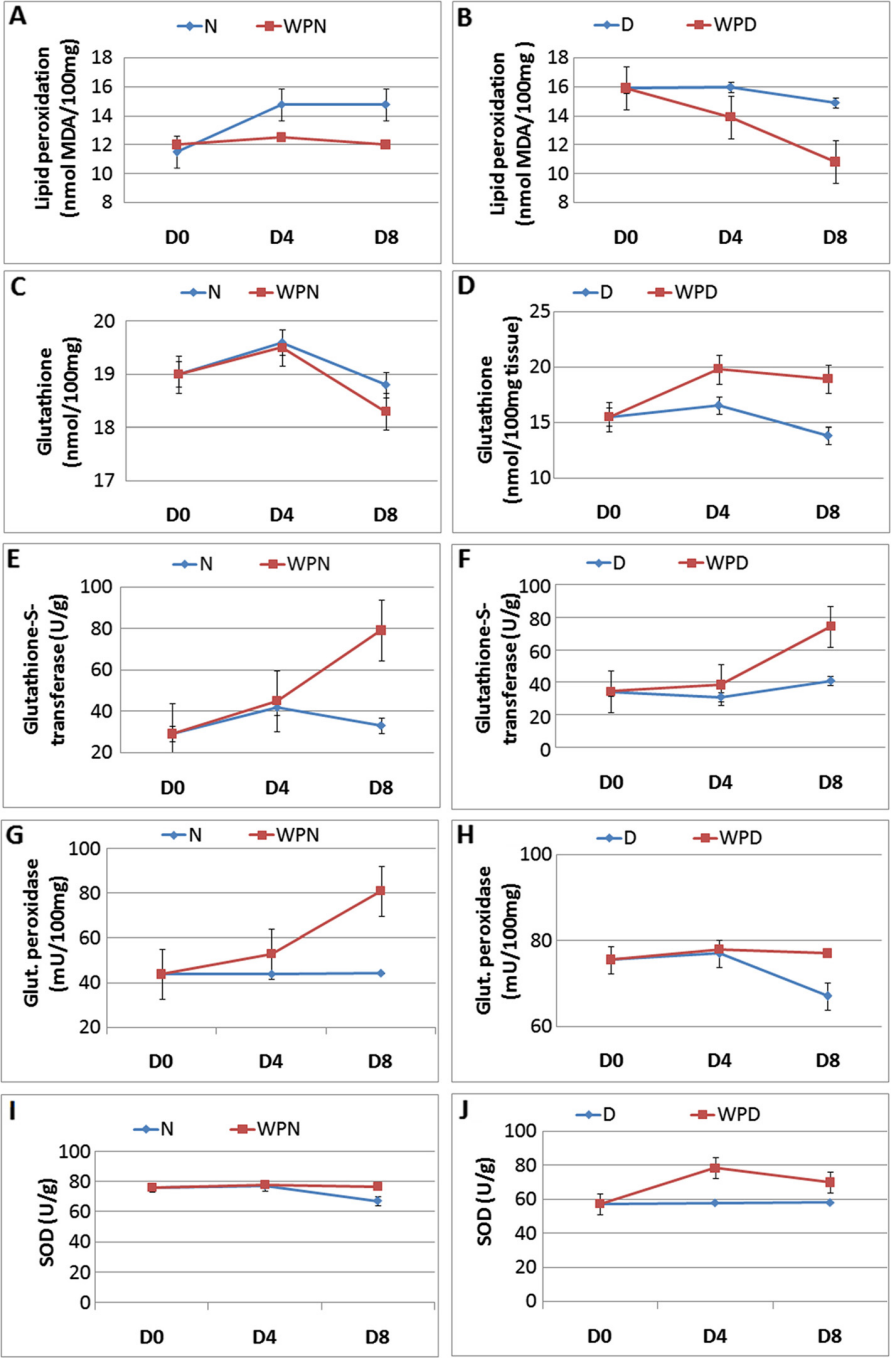
**Effect of whey protein administration on liver oxidative stress status (A,B) and antioxidant defense system (C-J) of wounded normal and wounded streptozotocin-induced diabetic rats.** WP was found to stimulate the antioxidant defense system by increasing the level of glutathione and the activity of glutathione-S-transferase, glutathione peroxidase and superoxide dismutase (SOD) in wounded non-diabetic and diabetic rats. Non-wounded N; wounded normal: N; Whey protein-treated wounded normal: WPN; Non-wounded diabetic: Non-wounded D; Wounded diabetic: D; Whey protein-treated wounded diabetic: WPD.

The liver lipid peroxidation increased in wounded non-diabetic, uninjured diabetic and wounded diabetic rats compared with uninjured controls. Of the treated groups, only wounded diabetic rats treated with WP for 8 days exhibited a significant decrease in comparison to the corresponding wounded diabetic control. Glutathione content in the liver was significantly decreased (p < 0.05; LSD) in diabetic rats 8 days after wounding, and the WP treatment of these animals produced a highly significant increase, returning the value closer to control ones. Glutathione-S-transferase activity in the liver was greatly increased due to 8 days of WP treatment in wounded non-diabetic and wounded diabetic rats. Glutathione peroxidase activity in the liver increased in non-diabetic treated rats 8 days after wounding compared with the corresponding wounded non-diabetic control. The uninjured and wounded diabetic rats exhibited a significant increase compared with the normal uninjured and wounded groups. Treating wounded diabetic rats with WP did not induce a significant effect compared with the corresponding wounded diabetic control. Liver SOD activity was significantly decreased in uninjured diabetic and wounded diabetic rats compared with wounded and uninjured non-diabetic rats. The treatment of wounded diabetic rats with WP produced a significant increase (p < 0.05; LSD) on the 4^th^ day after wounding and treatment. With regard to the ANOVA test, the effect between groups on glutathione content and SOD activity was significant (p < 0.05; F-prob.). Although the general effect on lipid peroxidation was highly significant, the effect on glutathione-S-transferase and glutathione peroxidase activities was very highly significant (Figure 8).

### Discussion and conclusions

Wound healing begins immediately after injury and proceeds by well-organized integration and interaction between different types of cells and tissues. Wound healing is characterized by centripetal movement of the wound edge toward the center of the wound, producing wound closure. The process of wound healing can be divided into five phases – the cellular phase (granulation), narrowing of the wound area (wound contraction), collagen deposition (collagenation), epithelial covering (epithelialization) and scar remodeling (cicatrization). These phases are overlapping, and any agent that accelerates the process promotes wound healing [33,34]. Diabetes mellitus delays wound healing by affecting these processes [35,36].

Examination of the wounds in this study demonstrated that although diabetes mellitus slows wound healing, daily WP administration after wounding hastens wound closure in both diabetic and non-diabetic rats. These results are consistent with those of Ebaid et al. [17], who revealed that WP supplementation improved both the healing and closure of diabetic wounds.

The enhancement of wound healing in WP-treated diabetic rats was concomitant with a potential improvement in the glycemic state and alleviation of the lowered serum insulin levels. Furthermore, non-diabetic rats that were orally administered WP daily for 4 or 8 days exhibited an enormous increase in serum insulin levels consistent with enhanced wound closure, although serum fasting glucose levels were not significantly affected. These results are in concordance with previous publications [37,38] indicating that WP has insulinotropic effects in non-diabetic and diabetic conditions. As reported in previous studies, WP contains many amino acids at varying concentrations [38]. Most of these amino acids are likely to act as insulin secretagogues after WP supplementation. It is well known that amino acids stimulate insulin release from pancreatic β-cells both alone and in combination [39-41]. The mechanism by which amino acids induce insulin release from β-cells is complex, and several metabolic pathways are activated, depending on the type of amino acid [38,40]. In addition to the insulinotropic action of amino acids, WP produced an increase in the plasma glucose-dependent insulinotropic polypeptide after ingestion [38]. In addition to the stimulatory effects of whey protein on insulin secretion, the present study revealed that the administration of whey protein to normal and diabetic wounded rats enhanced the proliferation and multiplication of β-cells which seemed to be more granulated in the treated animals. Thus, this also provides evidence that support the hypothesis that whey protein increased insulin levels in both normal and diabetic rats.

The direct positive effect of insulin on the enhancement of wound healing has been unclear in previous publications. However, many studies [42-44] found that insulin treatment by topical application or by injection accelerated wound healing. By contrast, Weringer et al. [45] revealed that there was no detectable difference in the duration of the healing response in either insulin-treated or non-treated diabetic mice.

Hyperlipidemia with or without diabetes mellitus may impair wound healing [46,47]. The diabetic rats in the present study exhibited a profound elevation in total cholesterol, triglycerides, LDL-cholesterol and vLDL-cholesterol levels. The cardiovascular risk indices, represented by the ratios of total cholesterol levels and LDL-cholesterol to HDL-cholesterol levels, were also remarkably increased. These changes may cause deterioration of the process of healing. Sen et al. [46] reported that re-epithelialization was reduced in hypercholesterolemic mice. Jang et al. [10] observed that hypercholesterolemia is associated with angiogenic impairment, which in turn delayed wound healing. WP treatment of wounded diabetic rats produced marked amelioration of the tested serum lipids and the calculated cardiovascular risk indices, which may be secondary to the improvement of serum insulin level. Based on our results and previous publications, it can be concluded that the alleviation of serum lipids and cardiovascular risk indices may be involved, at least in part, in the enhanced wound healing observed in diabetic rats treated with WP.

In our previous study [17], the early changes in inflammatory cytokines (IL-1β, TNF-α and IL-6) during the acute phase of the inflammatory response and the effect of WP were investigated at 6 and 24 h after wounding. The increase in these inflammatory cytokines as a result of neutrophil infiltration during the early phase after wounding is particularly essential for debridement and the clearing of infection by the absorption of wound exudates [6,48]. Neutrophils, which are the first cells to arrive at the wound, eliminate microorganisms and then undergo apoptosis. Afterward, neutrophils are rapidly and efficiently consumed by macrophages in a process that does not lead to further inflammation [36]. In the present study, we assessed the effect of WP on these inflammatory cytokines as well as on the anti-inflammatory cytokine IL-6, but during later periods, at 2, 4 and 8 days after wounding in normal and diabetic rats.

The serum TNF-α level was moderately increased in non-diabetic rats as a result of wounding, but it was strongly elevated in the wounded diabetic rats 4 days after wounding. The serum concentration of IL-1β was significantly increased in wounded non-diabetic rats on day 2 and 8 but was not significantly affected in wounded diabetic rats compared with the corresponding uninjured control. The serum IL-6 level was significantly increased in wounded non-diabetic rats, but it was not significantly affected in the wounded diabetic group. However, levels of the anti-inflammatory cytokine IL-10 in serum were significantly decreased in wounded non-diabetic rats but were not significantly affected in wounded diabetic rats. From these contrary results, it can be concluded that the deterioration of both inflammatory and anti-inflammatory cytokines in diabetic rats may play a crucial role in the delay of wound healing in diabetes mellitus.

WP treatment of wounded animals in this study did not significantly affect serum TNF-α, IL-6 and IL-10 levels after 2 days. When the treatment period was extended to 4 days, the levels of the inflammatory cytokines TNF-α, IL-1β and IL-6 decreased. The decrease in IL-1β and IL-6 levels continued as the treatment progressed for 8 days in both wounded non-diabetic and diabetic rats treated with WP, whereas the TNF-α concentration was unaffected in treated wounded diabetic rats after 8 days. The concentration of the anti-inflammatory cytokine IL-10 in serum was significantly increased after treatment with WP for 4 days, but it was not significantly altered after 8 days of treatment in both wounded non-diabetic and diabetic rats. Thus, it can be suggested that WP initially and acutely evokes the pro-inflammatory and inflammatory response at 6 and 24 hours [16] and then hastens the switch from inflammatory to anti-inflammatory responses during the process of wound healing. These findings agree with Peranteau et al. [49], who reported that overexpression of IL-10, an anti-inflammatory cytokine, decreases the inflammatory response to injury and creates an environment conducive to regenerative wound healing. Therefore, WP improved wound healing in diabetic mice because it significantly decreased the elevated pro-inflammatory cytokines (IL-1β, IL-6 and TNF-α) and increased IL-10 in the plasma and wound tissues. Based on these previously described ideas, it can be concluded that WP limited prolonged inflammation and modulated the immune response during the progress of wound healing in both normal and diabetic animals.

An increase in free radicals and diminished antioxidant activity may worsen the situation and account for the delay in wound healing in diabetic patients. In the present study, liver lipid peroxidation, which is an indicator of oxidative stress, was markedly increased in wounded non-diabetic and diabetic rats compared with the corresponding uninjured animals. This result is in accordance with Rosenbaum et al. [47], who suggested that oxidative stress, regardless of its source, induces cellular dysfunction in endothelial and smooth muscle cells and reduces angiogenesis and the healing process.

In the present study, the treatment of wounded diabetic rats with WP profoundly decreased the elevated lipid peroxidation and improved the antioxidant defense system by increasing hepatic glutathione levels and the activity of glutathione-S-transferase, glutathione peroxidase and SOD. These results agree with those of Ebaid et al. [17], who discovered an increase in GSH level and a decrease in reactive oxygen species (ROS) and hydroperoxide in the wounded tissue of diabetic rats after treatment with WP. This suggests that WP may improve wound healing, at least in part, by the modulation of oxidative stress and the antioxidant defense system.

In conclusion, WP hastens the wound healing process in diabetic rats by limiting prolonged inflammation, improving hyperglycemic and hyperlipidemic states, increasing insulin level, and amelioration of oxidative stress and the antioxidant defense system. However, we continue to perform further studies to assess the effect of WP on the phases of wound healing, including granulation, collagenation, epithelialization and cicatrization at the cellular and molecular levels.

## Abbreviations

IL-1b: Interleukin-1b; IL-6: Interleukin-6; IL-10: Interleukin-10; GP: Glutathione; MDA: Malondialdehyde; ROS: Reactive oxygen species; SOD: Superoxide dismutase; STZ: Streptozotocin; TNF-α: Tumor necrosis factor alpha.

## Competing interests

The authors declare that they have no competing interests.

## Authors’ contributions

HE designed the study, prepared figures, corrected and revised the manuscript. OM was responsible for data analysis and drafted the manuscript. AM was responsible for the animal model and the techniques. RA read and described histology. All authors read and approved the final manuscript.
